# Functional and Rehabilitative Outcomes of Patients Affected by Bone Cancer of the Upper Limb Treated with MUTARS Prosthesis: A Narrative Review

**DOI:** 10.3390/jcm13061651

**Published:** 2024-03-13

**Authors:** Sefora Codazza, Paola Emilia Ferrara, Adelaide Aprovitola, Mariantonietta Ariani, Fabiana La Cagnina, Daniele Coraci, Giorgio Ferriero, Gianpaolo Ronconi

**Affiliations:** 1 Department of Ageing, Neurosciences, Head-Neck and Orthopaedics Sciences, University Polyclinic Foundation Agostino Gemelli Scientific Hospitalization and Care Institutes, 00168 Rome, Italy; paolaemilia.ferrara@policlinicogemelli.it (P.E.F.); adelaide.aprovitola01@icatt.it (A.A.); mariantonietta.ariani01@icatt.it (M.A.); 2 Physical and Rehabilitation Medicine, University of Rome Tor Vergata, 00133 Rome, Italy; fabiana.lacagnina@students.uniroma2.eu; 3 Department of Neuroscience, Section of Rehabilitation, University of Padova, 35122 Padova, Italy; daniele.coraci@unipd.it; 4 Physical and Rehabilitation Medicine Unit, Maugeri Scientific Clinical Institutes (Scientific Hospitalization and Care Institutes), 21049 Tradate, Italy; giorgio.ferriero@uninsubria.it; 5 Department of Biotechnology and Life Sciences, University of Insubria, 21100 Varese, Italy; 6 Department of Rehabilitation, Catholic University of Sacred Heart, 00168 Rome, Italy

**Keywords:** megaprostheses, MUTARS, rehabilitation, functional outcomes, bone tumors, bone metastasis, upper limb, quality of life

## Abstract

Megaprostheses are well-known, reliable, and effective reconstruction prostheses used in oncologic surgery for limb salvage in patients affected by primary or metastatic bone tumors. Rehabilitation plays a major role after MUTARS replacement, with the aim of improving function after surgery and maintaining the highest possible quality of life. Only a few studies have been published about the use of megaprostheses for the upper limb. The aim of this narrative review is to describe the results of functional and rehabilitative outcomes of patients affected by bone primary or metastatic bone cancer of the upper limb and surgically treated with MUTARS prostheses. A comprehensive search was conducted on PubMed and Scopus using the following MESH terms: “Mutars”, “Megaprosthesis”, “bone”, “tumors”, “metastasis”, “upper limb”, “rehabilitation”, “outcome”, “quality of life”, and 10 studies were included. The most frequent oncological pathology was found to be metastases of the proximal humerus treated with modular endoprosthesis or modular reverse implants. Outcome measures used were ROM, MSTS, ASES, DASH, Constant-Murley score, Enneking score, VAS, MEP, TESS, and WOSI. Reconstruction of the proximal humerus with the MUTARS system seemed to be a valid treatment option after bone tumor resection. Rehabilitation after MUTARS surgery is very relevant, but currently, functional and rehabilitative outcomes are inadequately represented in the literature. Hence, further studies are needed to define standardized rehabilitation protocols after oncological orthopedic surgery that can be applied routinely in clinical practice.

## 1. Introduction

Bone cancer has a very negative influence on a patient’s quality of life; therefore, a multidisciplinary approach is fundamental to guarantee the best possible care. Bone tumor treatments have evolved during the past decades. The heterogeneity of tumors with regard to molecular genesis, histology, clinical characteristics, the plethora of treatments, and response to them makes the management of these neoplasms particularly challenging. Therefore, the complexity of care necessitates systematic multidisciplinary team management and should be performed in reference centers capable of providing access to the full spectrum of care, beginning with the diagnosis, involving oncologists, radiologists, and histopathologists, and then the treatment where not only the orthopedic surgeons play a main role, but also radiotherapists, psycho-oncologists, and oncologists. Nonetheless, rehabilitation programs are fundamental to address the disabilities caused by cancer and its treatment, to help patients and long-term survivors, to reduce morbidity, and to improve their quality of life.

In recent decades, there has been an increase in literature reports on musculoskeletal oncologic surgery, driven by the improved life expectancy of cancer patients, which has resulted in an increased risk of bone metastases [1].

Rehabilitation plays a major role in post-surgery recovery, aiming to maintain the highest possible quality of life through physical and psychological recovery and possibly a social state as closely as possible to the pre-existing one at the onset of the disease.

Primary bone tumors are uncommon, accounting for less than 0.2% of all malignancies [2], and the most common histologic varieties in the second decade of life are osteosarcoma and Ewing’s sarcoma and in older age chondrosarcoma [3,4].

On the other hand, bone is a particularly common site of metastases, especially from breast cancer and prostate carcinoma, followed by lung cancer and gastrointestinal and genitourinary tract tumors [5].

In the context of oncological pathologies of the upper limb, the humerus is the second most commonly affected long bone after the femur, with an incidence of humeral pathologic fractures ranging between 16 and 27% [6]. The proximal humerus is among the most commonly affected sites for metastases, characterized by a late presentation and extensive bone loss causing severe pain and impairment of daily activities, resulting in an overall decline in the patient’s quality of life [6].

The elbow is a rare site for bone and soft tissue tumors, with a reported incidence of <1%; however, distal upper arm lesions often require surgical resection around the elbow, with nerve and muscle sacrifice, resulting in functional disability [7].

Acrometastases, which are metastases located distal to the elbow, should also be mentioned; these bony lesions in hands are a very rare occurrence, representing 0.1% of all metastatic bone lesions, and they are usually associated with advanced cancer, with a poor prognosis [1].

Different reconstructive techniques for the upper limb oncologic disease have been described over time, with the aim of achieving local tumor control, reducing pain, providing acceptable joint stability, and improving function in the affected extremity [8].

There are different surgical approaches to replacing proximal humerus after a wide resection: biological reconstructions (allografts and autografts), prosthetic reconstructions (anatomic endoprostheses, total reverse shoulder prostheses, and megaprostheses), graft-prosthetic composite reconstructions, clavicular pro humerus, and arthrodesis. There is currently no unique line of thinking, but according to the literature, biologic reconstructions are indicated mainly for young patients with a long life expectancy and high functional demands, while prosthetic reconstructions represent a reliable option, especially for patients with medium or short life expectancy who could still maintain decent upper limb functionality with a reasonable recovery time [6].

The main goal of oncological surgery is limb salvage and best possible function preservation; however, this is not always possible due to the large tissue and joint resection often needed to eradicate the tumor [9].

During the last 4 decades, the use of megaprostheses, also known as MUTARS prosthesis (Modular Universal Tumor and Revision System), has radically revolutionized the surgical management of patients with malignant bone tumors.

Megaprostheses are modular endoprostheses, consisting of several different components in promptly available sets, which can be assembled in different combinations to fit the specific skeletal defect allowing limb sparing. The rational use of this system aims to restore the function of limbs as quickly as possible in order to improve quality of life, minimize the morbidity associated with the surgical procedure and hospitalization, and continue oncologic therapies [10].

The modularity of MUTARS makes it versatile for the reconstruction of different skeletal segments, meeting the biomechanical requirements of the single surgical case. Compared with other reconstruction methods, MUTARS have some advantages: relatively simple, early weight bearing, fewer early complications, and a lower rate of mechanical failures [11]. Moreover, the functional outcome after reconstruction with megaprostheses is often very satisfying, and patients can enjoy a good quality of life [12].

Modular endoprostheses for the lower limb have been nearly dominanting surgical practice since the1980s, with consistent evidence from the literature [13]; instead, little information is available about the modular endoprosthetic system for the replacement of the tumors of the upper limbs [14]. The rehabilitation programs and functional recovery aspects are closely linked to the surgical technique, the width of the resection, the type of prosthesis and its biomechanics, the characteristics and staging of the tumor, and the age and clinical features of the patient.

Functional impairment of the upper limb and instability of the shoulder represent a major challenge in the rehabilitation program after oncologic surgery. This loss of comfort and ability in active movements is caused by the undesirable translation of the prosthesis, resulting from decreased centering in the glenoid [9], and depends on the integrity of the rotator cuff muscles, tendon insertions, or the axillary nerve, which could be sacrificed to achieve satisfactory tumor resection.

To adapt and develop an adequate post-surgical rehabilitation program, it is important to note that for the upper limb, the most commonly involved site in tumors of the proximal humerus, the standard treatment is considered to be a wide resection of up to 10 cm, beyond which worse functional outcomes are reported [9,15]. This large shoulder resection leads to the need for a complex reconstruction in order to restore upper limb functionality, and it could be obtained by using different types of MUTARS implants, which allow for the re-anchoring of any tendon that did not need removal due to tumoral involvement, even though they are widely manipulated during the excision phase of the surgical technique [9].

The improvements in diagnostic technologies and oncologic therapies allow an early-stage detection of bone tumors enabling surgical radicalization. In addition, with advances in protocols and oncological treatment of cancer, there is an increase in the survival rate of patients with disability and functional impairment in the affected arms.

The aim of this narrative review is to describe the short- and long-term functional and rehabilitative outcomes of patients affected by bone primary or metastatic bone cancer in the upper limb and surgically treated with MUTARS prostheses.

## 2. Methods

All studies published in PubMed and Scopus between January 2010 and June 2023 were included in the study, after applying the specific search terms listed below.

For this narrative review, we included original articles on modular tumor endoprostheses of the upper limb published in English in PubMed- and Scopus-indexed journals as eligible. All the papers not related to upper limb bone tumors or non-modular prosthetic implants were excluded.

A comprehensive search was conducted on PubMed and Scopus using the following MESH terms: “Mutars”, “Megaprosthesis”, “bone”, “tumors”, “metastasis”, “upper limb”, “rehabilitation”, “outcome”, “quality of life”, in human, in oncological disease without other limits.

Reviews, case reports, duplicates, and meta-analyses were excluded manually.

All articles with “case report” in the title were excluded in the first step of article selection. From all articles finally eligible for this study, full-text articles were downloaded from the respective journal’s website. Those studies finally eligible for the study were reviewed by both the first author (SC) and senior author (PEF).

No additional analyses were performed. After the removal of duplicates and review by title and abstract, 13 full-text articles were assessed for eligibility and 10 studies were included in descriptive synthesis. The type of observational study was retrospective for all the articles [9,16,17,18,19,20,21,22,23], except for one which is prospective [24].

Data extracted from the selected studies were patients’ characteristics (number, mean age, sex), bone tumors of the upper limb (histological characteristics and distribution between primary and metastatic lesions), the models of prosthesis used for surgical replacement, the outcome measures, and the timing (the years retrospectively analyzed or the timing of mean time of follow-up), and the main results are summarized in Table 1.

## 3. Results

The analysis of the selected papers about functional outcomes of patients affected by upper limb primary and/or metastatic bone tumors treated with MUTARS is summarized in Table 1.

The retrospective analysis of the studies selected covers a period between 1970 and 2022. A total of 359 patients are included, everyone with primary and/or metastatic bone tumor of upper limbs surgically treated with modular prostheses, localized in the elbow in one paper [20] and in the shoulder in all the other articles considered. The age of patients ranges from 7 to 86 years, with females being more represented than males in the papers considered.

Bone tumors reported were 177 metastatic, 59 osteosarcoma, 43 chondrosarcoma, 6 soft tissue sarcoma, 3 liposarcoma, 7 Edwings’ sarcoma, 3 giant cell tumors, and 63 other tumors.

Regarding the type of MUTARS implant, authors reported endoprosthesis replacement in the proximal humerus in five papers [9,16,17,18,23] and inverse proximal humerus endoprosthesis in four articles [17,19,21,22]. Only one paper studied modular prostheses of the elbow [20].

The range of follow-up after surgery was between 3 months and 29 years in the different retrospective studies.

Outcome measures used were Musculo-Skeletal Tumor Society (MSTS) in 9 studies [9,16,17,18,19,20,21,22,23], shoulder/elbow Range Of Motion (ROM) in 5 articles [17,19,20,21,24], American Shoulder and Elbow Surgeons score (ASES) in 2 studies [22,23], Disabilities of the Arm, Shoulder, and Hand score (DASH) in 2 studies [9,19], Constant-Murley score in 2 papers [19,22], Enneking score [24], Visual Analog Scale (VAS) for pain [19], Mayo Elbow Performance (MEP) [19], Toronto Extremity Salvage Score (TESS) [20], and Western Ontario Shoulder Instability Index (WOSI) [9] in 1 study.

## 4. Discussion

The goal of this narrative review is to describe the short- and long-term functional and rehabilitative outcomes of the patients affected by primary or metastatic bone tumors in the upper limb surgically treated with MUTARS prostheses.

The data collected from the retrospective studies of upper limb oncologic surgery included in this narrative review are focused mostly on the orthopedic goals to improve the surgical techniques or to reduce the complications, and the rehabilitative aspects are very poorly represented.

The results of the retrospective analysis seemed to be heterogeneous from multiple points of view, showing considerable variability regarding various constitutive elements. The first observation is that the demographic characteristics of the sample possess a mean age ranging from a minimum of 26 to a maximum of 61.5 years, so the review covers both pediatric and adult age, with extreme age values ranging from 7 to 86 years; the gender distribution was not always homogeneous, with a slight predominance of the female sex. Also, the sample size of the various studies appeared variable, ranging from a minimum of 10 to a maximum of 85 subjects.

Secondly, the types of bone tumors in the studies were also heterogeneous, both in histological terms and in terms of primary or secondary nature: two papers [21,23] analyzed only primary tumors, two articles studied only metastatic lesions [9,16], while most studies collected data regarding both primary and secondary bone lesions [17,18,19,20,22,24]. Regarding the tumor anatomic site, only one paper analyzed the elbow [20], while the remaining articles studied the shoulder, and this distribution is consistently in line with the evidence available in the literature, according to which the proximal humerus is the site most commonly involved in bone tumors, unlike the elbow which is rarely affected [6].

Regarding outcome measures, all articles measured ROM and function using different assessment scales, although the most used was the MSTS [25]. The active range of motion in degrees was generally recorded for shoulder flexion, abduction, and external rotation with the hanging arm in a neutral position and elbow flexion of 90°.

Three articles [9,16,23] do not use the ROM for post-surgical joint assessment: Klingebiel et al. [23] combined two functional outcome measures, the MSTS score with the ASES, which does not specify the degrees of ROM but rather categorizes the shoulder as either pseudo-paralytic or essentially normal; and El Motassime et al. [9] associated MSTS score with DASH [26] and WOSI [27] scores, with no specific ROM measurement.

Lastly, the timing of observation and the mean follow-up after surgery were also very heterogeneous. The observation time varied from a minimum of 2 to a maximum of 23 years, while the mean follow-up showed a range of average values from 6 to 38 months after surgery.

The main problem after segmental tumor resection of the proximal humerus is the marked impairment of active movements of the shoulder, which depends not only on the loss of huge parts of the shoulder girdle musculature and its bony attachment but also largely on the type of prosthesis used for anatomical replacement.

Many different prosthetic systems for defect reconstruction are currently used for bridging humeral resection, although nowadays too little information is still available about the modular oncologic replacement for the upper limb due to a malignant lesion [28].

Different MUTARS implants with a cemented or uncemented fixation manufacture have been described in the included articles for bridging the resulting anatomic defects and can essentially be grouped into three categories: endoprosthesis [9,16,21,23,24], total endoprosthesis [18], and inverse endoprosthesis [17,19,22].

Endoprosthetic reconstruction is described to have significative advantages like relative ease of insertion, fewer procedure-related complications, early return to function, and high implant survival. Functional outcomes are strictly related to the preservation and integrity of the abductor apparatus [29].

However, the main disadvantages of endoprosthetic replacement are the limitation of the post-surgical active movement of the shoulder and a considerable risk of implant instability, caused by difficulties in soft tissue reconstruction [14].

Regarding ROM, our data confirmed that the active range of movement after replacement is globally sacrificed. In fact, Raiis et al. [24] observed a mean shoulder flexion of 34° (range 0–90°), abduction of 33° (range 0–90°), and external rotation of 12° (range 10–50°). Also, Goryń et al. [21] found very limited results of active flexion and abduction in patients after surgery.

However, patients with a complete rotator cuff repair showed a significantly better range of motion compared to patients with a partial or no repair [24], underlining the importance of the integrity of the rotator cuff from a functional point of view.

No evidence of implant loosening was observed for patients treated with proximal humerus replacement with endoprosthesis; however, stress shielding, defined as new-onset bone resorption at bone/prosthesis interface, is common after shoulder reconstruction with modular megaprosthesis and might be associated with mechanical failures [30]. This eventuality has a great relevance in the rehabilitative field, because the necessity of revision surgery procrastinates the rehabilitation programs, lengthens recovery times, and could affect functional outcomes.

Also, anatomic reconstruction of the proximal humerus with a total endoprosthesis performed for tumor resection has been used to maintain cosmetic appearance and shoulder stability, but shoulder function has been limited, with an active elevation not exceeding 60° in the sagittal, coronal, or scapular plane [18].

About implant stability, El Motassime et al. [9] interestingly highlighted how MUTARS endoprosthetic replacement may be associated with a reduction in function and shoulder firmness stability. This loss of comfort depends on the undesirable translation and misalignment of the joint relationship between the prosthesis and the glenoid, due to the surgical involvement and manipulation of the structures that normally stabilize the shoulder. Especially when rotator cuff muscles or tendon insertions or the axillary nerve have to be sacrificed to obtain a satisfactory tumor margin, patients who reported shoulder instability showed lower scores of MSTS, DASH, and WOSI than those who reported having stable shoulders, probably also for the apprehension that led to a limited active movement of the joint, and thus obtained worse functional results.

Piccioli et al. [16] demonstrated how MUTARS replacement is particularly useful in cases of metastasis of the humeral metaphysis, where the cortical bone has lower rigidity and osteosynthesis would not be recommended due to the risk of surgical failure. Modular endoprosthesis provided satisfactory functional outcomes with significant results in hand position, manual dexterity, and lift ability, especially in the case of preservation of the deltoid.

Reverse shoulder arthroplasty is an established procedure in older patients with severe osteoarthritis with deficient rotator cuff [31]. However, due to the increase in the incidence of bone tumors in recent years [32], this type of surgical replacement was recently introduced for the surgical management of younger patients with primary or metastatic malignant lesions of the proximal humerus with major loss of muscles, althoughonly a few reports are available in the literature about inverse shoulder replacement [33].

Reverse implants are characterized by high stability and good functional results, because the center of rotation is shifted medially and distally, increasing the lever arm of the deltoid muscle, thus allowing the recruitment of more anterior and posterior deltoid fibers [14].

About post-surgical active motility, our results showed that inverse shoulder implants can improve the active range of motion significantly in comparison to endoprosthesis [21,23,24] and anatomically shaped prostheses [18].

All three studies on the implantation of an inverse tumor prosthesis after segmental resection of the proximal humerus showed a significative improvement in the active range of motion in the restored shoulder joint [17,19,22], confirming the evidence from the literature that shoulder range of motion and functional results have improved significantly since the introduction of the reverse shoulder prosthesis for the treatment of proximal humerus tumors [33].

In addition, we noticed a correspondence between the post-surgical articular assessment and the functional evaluation in inverse shoulder replacement. The satisfactory mean shoulder ROM values showed an overall correlation with MSTS, used in all the studies [17,19,22], and also with Constant, DASH [19], and ASES [22]. Contrarily, we have not found coherence between ROM and function for anatomical prostheses, which globally had limited shoulder active movements, in the presence of functional scales such as the Ennekig [24] and the MSTS [23] with high scores.

The function of the affected limb was acceptable, even though shoulder elevation was restricted, and this may be due to the fact that the elbow and hand function is regularly intact, providing an adequate use of the arm in daily activities represented by satisfying MSTS scores between 69 and 79% [17]. These data confirmed the observations previously reported by Cannon et al. [34] about the supporting action of the distal articular districts. That replacement of the proximal humerus provides an excellent platform for the function of the ipsilateral elbow and the hand, with a very satisfying manual dexterity.

Regarding this mismatch, some authors have observed that the comparability between conventional endoprostheses and the inverse implants using the MSTS scoring system is not feasible as the severe restrictions of the shoulder function are not reflected by this score [17].

Another fundamental difference between anatomical and reverse prostheses is the influence of the integrity of the axillary nerve on functional outcomes. According to Streitbuergeret al.’s study [17], the integrity of the axillary nerve is the key factor for the functional outcome of inverse prostheses, because it allows a medium active abduction of80° and 84° elevation, confirming that the reverse implant offers a significant improvement in active shoulder function in patients in whom the deltoid function can be preserved in comparison to anatomically shaped prostheses. On the other hand, the preservation of deltoid innervation seems to be non-significant using anatomical prostheses, as the preserved function of the deltoid did not seem to improve active ROM, which is however limited by the nature of the implant [14]. So, for patients with total or partial deltoid impairment, the use of inverse arthroplasty is not recommended, because there is no benefit regarding an improved active shoulder ROM [17,24], as reported in the recent literature [14].

Modular or custom-made elbow megaprostheses have been recently proposed in traumatology as an alternative to osteosynthesis or conventional prosthesis for managing comminuted articular fractures in elderly patients with poor bone stock [35]. Casadei et al. [20] showed that modular prosthesis could yield good functional outcomes in elbow reconstruction for both primary and metastatic lesions; nerve palsy is the most common complication but is often temporary and does not seem to affect ROM and postoperative function.

The functional results of MUTARS prostheses of the included studies of our narrative review were excellent or good in most of cases. Although the risk of complications with megaprosthetic implants seemed to be relatively low, infections remain the worst and most frequent cause of implant failure, often leading to revision and implant explantation [36]. Reasons for these high infection rates are the long surgery time, the large incisions, and the immunosuppression due to chemotherapy and radiotherapy, as well as the increasing resistance of the bacteria against antibiotic drugs.

Several preventive procedures have been evaluated to decrease the risk of contamination of the implants, such as hygienic precautions and hydrophilic materials to minimize bacterial adhesion and impregnation with antiseptics and antibiotics. In addition, new materials like iodine and silver are demonstrating encouraging results in reducing the risk of infections. Several pieces of evidence in the literature support the notion that silver-coated prostheses are associated with lower infection rates, with the combination of a high degree of antimicrobial activity and a low level of human toxicity [37].

Comparing MUTARS with non-modular prostheses, we can notice an important difference in post-surgical function and range of motion. MUTARS reverse total shoulder arthroplasty [17,18,19,22] has better outcomes versus endoprosthesis [21,23,24] and anatomically shaped prostheses [18]. On the contrary, it is reported in the literature that patients who underwent anatomic total shoulder arthroplasty generally had better functional outcomes compared to those who underwent reverse total shoulder arthroplasty, which showed reduced upper limb proximal mobility [38].

## 5. Conclusions

Megaprostheses are well-known, reliable, and effective reconstruction tools in oncologic surgery used for limb salvage in patients affected by primary or secondary bone tumors. The use of MUTARS tumor endoprostheses in the lower limb is well documented; to our knowledge, only a few studies have been published on the use of this modular system for the treatment of large bone defects in the upper limb.

Many different modular endoprosthetic systems for defect reconstruction of the proximal humerus are currently in clinical use, and each of them has its own advantages and disadvantages, with an important impact on the post-surgical rehabilitation program.

Endoprosthetic replacement is characterized by ease of insertion, low complication rates, and high implant survival but shows a considerable risk of implant instability.

Reverse shoulder arthroplasty is characterized by high implant stability, better active post-surgical range of motion, and good functional results but requires at least a partially preserved function of the abductor apparatus, to avoid implant dislocation.

Rehabilitation after MUTARS prosthesis replacement is very relevant, but currently, functional and rehabilitative aspects are inadequately represented in the literature. So further studies with larger samples and clearer and more precise rehabilitative goals are necessary to define standardized rehabilitation protocols after oncological surgery, in order to introduce guidelines that can be routinely applied, with the aim of improving oncologic patients’ functionality and quality of life.

## Figures and Tables

**Table 1:** Description of the included studies.

Author (Years)	Type of Study	Patients	Bone Tumor	MUTARS	OutcomeMeasures	Follow-Up (Mean Range)	Results Mean (Range)
Raiss et al. (2010) [24]	Prospective	n:39 M/F:19/20 Meanage (range):60 (24–84) years	n:30 metastases n:9 malignant bone tumors	Endoprosthesis	Ennekingscore, ROM	1995–2008 months:38 (3–138)	Enneking score: 19 (7–27) Shoulder ROM: flexion 34° (0–90°), abduction 33° (0–90°) external rotation:12° (10–50°).
Piccioli et al. (2010) [16]	Retrospective	n:85 M/F:35/50 Meanage(range): 59 (45–80) years	n:87 metastases	n:30 endoprosthesis n:57 intra-medullary locked nailing	MSTS	2003–2008 months:8 (3–22)	MSTS: Endoprosthesis 73% Intra-medullary locked nailing: 79.2%
Streitbuerger et al. (2014) [17]	Retrospective	n:18 M/F:11/7 Meanage:42 (14–70) years	n:1 giant cell tumor n:11 sarcoma n:5 metastases	IPHP	MSTS, ROM	nr months:33 (10–120)	Axillary nerve preserved vs. not: 78% vs. 22% MSTS: 24.6/30 Active arm abduction 78°, active arm elevation 88° in patients with intact axillary nerve function
Wafa et al. (2015) [18].	Retrospective	n:34 M/F:10/24 Meanage (range) 26 (7–86) years	n:15 osteosarcoma n:7 chondrosarcoma n:7 Ewing’s sarcoma n:3 metastases n:3 liposarcoma n:1 giant cell tumor	n:24 THER n:10 expandable THER n:2 THER with reverse shoulder design	MSTS, ROM	1970–2012 years 8.2 (3 months–29 years)	MSTS: 83% (range, 60–93%) ROM <60° in sagittal, coronal, or scapular plane
Guven et al. (2016) [19]	Retrospective	n:10 M/F:5/5 Meanage (range):49.4 (31–70) years	n:8 chondrosarcoma n:1 metastasis n:1 multiple myeloma	n:3 RSTP n:4 SMR n:3 METS n:3IPHP	DASH, Constant-Murley, ROM, VAS, MSTS	2012–2014 months:18.2 (6–27)	Shoulder: flexion: 96 (30–160), abduction 88 (30–160), External rotation 13 (0–20). Constant Murley: 53.7% DASH: 26.2 VAS: 1.3 MSTS: 78.1%
Casadei et al. (2016) [20]	Retrospective	n:47 M/F:nr Meanage (range):nr	n:30 primary tumors n:17 metastases	n:25 MP n:22 SP	MEP, MSTS, TESS, ROM	1990–2012 months: 35 (7–168)	Elbow flexion ROM: - Primary tumor: 70° - Metastasis: 40° - MEP: 84% MSTS: 22/30 (73%) TESS: 70%
Goryń et al. (2017) [21]	Retrospective	n:11 M/F:6/5 Meanage(range):39 (23–78) years	n:5 osteosarcoma n:2 chondrosarcoma n:4 giant cell tumor	n:10 Endoprosthesis n:1 Arthroplasty	MSTS, ROM	2011–2015 months: 31 (8–65)	MSTS: 74%. None of the patients, except one, were able to abduct the shoulder more than 20°
Trovarelli et al. (2019) [22]	Retrospective	n:22 M/F:9/13 Meanage(range):55 (18–71) years	n:10 chondrosarcoma n:6 metastasis n:4 multiple myeloma n:2 giant cell tumors	RTSA	MSTS, Constant-Murley, ASES, ROM	2011–2018	MSTS: 29 Constant score: 61 ASES: 81 Shoulder ROM: abduction 103° (40–180), flexion 117° (40–180)
Klingebiel et al. (2021) [23]	Retrospective	n:65 M/F:40/25 Meanage(range):18 14–42) years	n:28 osteosarcoma n:16 chondrosarcoma n:11 Ewing’s sarcoma n:5 malignant fibrous histiocytoma n:5 others	Endoprosthesis	ASES, MSTS	2000–2019 months: 36 (18–80)	MSTS and ASES scores did not correlate with the extent of stressshielding
El Motassime et al. (2023) [9]	Retrospective	n:28 M/F:13/15 Meanage (range):61.5 (nr) years	n: metastasis	Endoprosthesis	MSTS, DASH, WOSI	2014–2022 Mean follow-up: 6 months	Significant better results in pain, function, and emotional acceptance in all outcome measures

MUTARS: Modular Universal Tumor and Revision System; ROM: Range Of Motion; MSTS: Musculoskeletal Tumor Society; IPHP: inverse proximal humerus endoprosthetic; THER: total humeral endoprosthetic replacement; RSTP: reverse shoulder tumor prosthesis; SMR: stemless modular reverse; DASH: disabilities of the arm, shoulder, and hand; VAS: Visual Analog Scale; nr: not reported; MP: modular prosthesis; SP: standard prosthesis; TESS: Toronto Extremity Salvage Score; MEP: Mayo Elbow Performance; RTSA: reverse total shoulder arthroplasty; ASES: American Shoulder and Elbow Surgeons; WOSI: Western Ontario Shoulder Instability Index.

## Data Availability

Data is contained within the original articles; further inquiries can be directed to the corresponding authors.
